# *In vitro *migration of cytotoxic T lymphocyte derived from a colon carcinoma patient is dependent on CCL2 and CCR2

**DOI:** 10.1186/1479-5876-9-33

**Published:** 2011-03-30

**Authors:** Klara Berencsi, Pyapalli Rani, Tianqian Zhang, Laura Gross, Michael Mastrangelo, Neal J Meropol, Dorothee Herlyn, Rajasekharan Somasundaram

**Affiliations:** 1 The Wistar Institute, 3601 Spruce Street, Philadelphia, PA 19104, USA; 2 Department of Medical Oncology, Thomas Jefferson University, 1015 Walnut Street, Philadelphia, PA 19107, USA; 3 Department of Medical Oncology, Fox Chase Cancer Center, 333 Cottman Avenue, Philadelphia, PA 19111, USA; 4 Division of Hematology and Oncology, University Hospitals Seidman Cancer Center and Case Western Reserve University, 11100 Euclid Avenue, Lakeside 1200, Cleveland, OH 44106-5065, USA

## Abstract

**Background:**

Infiltration of colorectal carcinomas (CRC) with T-cells has been associated with good prognosis. There are some indications that chemokines could be involved in T-cell infiltration of tumors. Selective modulation of chemokine activity at the tumor site could attract immune cells resulting in tumor growth inhibition. In mouse tumor model systems, gene therapy with chemokines or administration of antibody (Ab)-chemokine fusion proteins have provided potent immune mediated tumor rejection which was mediated by infiltrating T cells at the tumor site. To develop such immunotherapeutic strategies for cancer patients, one must identify chemokines and their receptors involved in T-cell migration toward tumor cells.

**Methods:**

To identify chemokine and chemokine receptors involved in T-cell migration toward CRC cells, we have used our previously published three-dimensional organotypic CRC culture system. Organotypic culture was initiated with a layer of fetal fibroblast cells mixed with collagen matrix in a 24 well tissue culture plate. A layer of CRC cells was placed on top of the fibroblast-collagen layer which was followed by a separating layer of fibroblasts in collagen matrix. Anti-CRC specific cytotoxic T lymphocytes (CTLs) mixed with fibroblasts in collagen matrix were placed on top of the separating layer. Excess chemokine ligand (CCL) or Abs to chemokine or chemokine receptor (CCR) were used in migration inhibition assays to identify the chemokine and the receptor involved in CTL migration.

**Results:**

Inclusion of excess CCL2 in T-cell layer or Ab to CCL2 in separating layer of collagen fibroblasts blocked the migration of CTLs toward tumor cells and in turn significantly inhibited tumor cell apoptosis. Also, Ab to CCR2 in the separating layer of collagen and fibroblasts blocked the migration of CTLs toward tumor cells and subsequently inhibited tumor cell apoptosis. Expression of CCR2 in four additional CRC patients' lymphocytes isolated from infiltrating tumor tissues suggests their role in migration in other CRC patients.

**Conclusions:**

Our data suggest that CCL2 secreted by tumor cells and CCR2 receptors on CTLs are involved in migration of CTLs towards tumor. Gene therapy of tumor cells with CCL2 or CCL2/anti-tumor Ab fusion proteins may attract CTLs that potentially could inhibit tumor growth.

## Background

Chemokines play an important role in immune homeostasis and immune surveillance (reviewed in [1-3]). Studies have demonstrated that chemokines influence immune reactions by regulating trafficking of dendritic cells (DC) and lymphocytes [4]. In tumor bearing individuals, the role of chemokines is paradoxical. Chemokines produced by tumor cells are known to stimulate autocrine tumor growth, progression and metastasis [4-10]. In contrast, chemokines produced by tumor cells can also attract chemokine receptor (CCR)-positive leukocytes into the tumor area, potentially leading to tumor growth inhibition *in vitro *and *in vivo *[9,11-13]. In colorectal carcinoma (CRC) patients, T-cell infiltration has been shown to be associated with good prognosis (reviewed in [14] and [15-19]). In these studies, favorable prognosis was correlated with the presence of tumor cells secreting chemokines such as CCL5, CXCL10 and CXCL1 that played a role in recruitment of CCR5^+^/CXCR3^+ ^T-helper (Th)1 cells or CX3CR1^+^, perforin^+^/granzyme B^+ ^T cells [17], [18]. Colorectal and pancreatic carcinoma cells are known to secrete CCL2 which is associated with increased tumor infiltration of macrophages [20-22]. However, there are mixed reports of good and bad prognosis due to increased infiltration of tumor associated macrophages in these studies [20-22]. In those studies, the level of tumor derived-CCL2 and its influence on T cell infiltration of tumor cells is unclear. In mouse tumor model system, there are indications that melanoma cells secreting high amounts of CCL2 attract macrophages resulting in tumor growth inhibition [23]. In another study, transfection of mouse CT-26 CRC cells with CCL2 gene resulted in decreased metastasis and increased susceptibility of tumor cells to macrophage lysis [24]. Thus, selective modulation of chemokine activity at the tumor site could attract immune cells resulting in tumor growth inhibition (reviewed in [25] and [1]). In mouse systems, *ex vivo *transduction of chemokines into tumor cells has provided potent tumor vaccines inducing tumor rejection, which was mediated by infiltrating T cells at the vaccine site. Infiltration of T cells into the tumor area was followed by rejection of both transduced and non-transduced tumor cells [11-13]. CD4^+ ^T-cell subsets have been implicated in tumor rejection induced by vaccination of mice with CCL19-transduced tumor cells [26], and CD8^+ ^CTL were instrumental in tumor growth rejection in mice following intratumoral delivery of CCL20 or CXCL12 via adenovirus vectors [27] or injection of CCL16-expressing tumor cells. Both CD4^+ ^and CD8^+ ^T cells were required for tumor growth inhibition to occur in mice injected intratumorally with CCL21 [28].

It has been suggested that immunological intervention of cancer patients has been largely unsuccessful due to limited ability of T cells to infiltrate tumors in vivo ([29,30]). Chemokines fused to anti-tumor Ab may be utilized to attract adoptively transferred tumor antigen (Ag) -specific T cells to the tumor site [31]. To develop immunotherapeutic strategies for cancer patients based on chemokines and their receptors, similar to the approaches already successfully used in mice, one must identify chemokines and their receptors involved in T-cell migration toward tumor cells.

Recently, we have shown in an organotypic culture system (reconstruct) that migration of CTL derived from a CRC patient towards autologous tumor cells was mediated by chemokine receptor CXCR3 expressed by the T cells, and CXCL11 chemokine secreted by the autologous tumor cells [32]. In the present study, we show that migration of CTL derived from another CRC patient is dependent on CCL2 and CCR2.

## Materials and Methods

### Cell lines

CTL 007, CTL020, CRC cell line (WC007) and fetal colon fibroblast cell line (FCFB/1) were established and maintained in culture as previously described [32,33]. Ten additional primary tumor tissues were obtained from CRC patients of various disease stages whose T cells were analyzed for chemokine receptor expression (data from 4 patients whose T cells are positive for CCR2 are shown in Table 1). Blood and tissue specimens were obtained in compliance with Helsinki Declaration with informed consent approved by Institutional Review Board of Thomas Jefferson University Hospital, Fox Chase Cancer Center and The Wistar Institute (Approval number 2109169).

**Table 1:** CCR2 expression by T-cell lines established from tumor infiltrating lymphocytes of CRC tissues.

Patient^a^		T cell phenotype	Expression of CCR2 (% positive cells)^b^
		
#	Dukes' Disease Stage		
296674	A	CD4	58.8
298884	B	CD4/CD8	68
1003485	B	CD4/CD8	48.2
05193	B	CD4/CD8	27.4

^a^Representative data from 4 patients whose T cells were positive for CCR2 expression.

^b^CCR2 expression determined by FACS analysis. Data represented are from a single experiment and the Results were confirmed in at least two independent experiments.

### Reagents

The following monoclonal antibodies (mAb) were used: mAb Nok-1 to Fas ligand (BD-PharMingen, San Diego, CA); mAb CH-11 to CD95 and anti-CD11a mAb (Immunotech, Westbrook, ME); fluoresceinated (FITC) or phycoerythrin (PE) -conjugated anti-CD:4, 8, 25, 29, 40, 40L, 44, 49a, 49b, 54 and 80; anti-CXCL-11 mAb; anti-human CCR:1, 2, 3, 5, 6, 7 and 9 mAb; anti- CXCR:1, 2, 3, 4, 5 and 6 mAb (R&D Systems, Minneapolis, MN); anti-human CCR:4, 8, and 10 mAb (Imgenex, San Diego, CA); anti-CCR11 and -CX3CR1 polyclonal Ab (Abcam, Cambridge, MA); FITC conjugated goat anti-mouse IgG (Invitrogen, Carlsbad, CA). Recombinant human CXCL11 was purchased from R&D Systems.

### Chemokine determination by RT-PCR or ELISA

mRNA was extracted from CRC cells (5 × 10^6^) using Fast Track 2.0 mRNA isolation kit (Invitrogen). The primers used were 5'-GCC CGG TGT CAT CTT CCT AAC CAA GC-3' and 5'-AGG GGA CAG GGG AAC TCT CAG AGC AA-3' for CCL3; 5'-TGC TGC TTT TCT TAC ACC GCG AGG AA-3' and 5'-AGA AGG GAC AGG AAC TGC GGA GAG GA-3' for CCL4; 5'-TCT GCA GCA CTT CTG TGT CTG-3' and 5'-GGA TCC TAG AAG GAG CTG GA-3' for CCL7; 5'-CAG TCC ATG AGA AGG AGT CCA-3' and 5'-AGA TCC TGC ACA GGA CTG TG-3' for CCL8; 5'-AGG GCA TGG GTT TTA TTA TAT ATA TAT-3' and 5'-TTT AAA AAT AAC TGA TAT TCA TGG-3' for CCL11; 5'-TCA TCT TTC CAC AAT AAC ATA TTT A-3' and 5'-GTT TAT TTG AGT ATT GCT GAT CTT T-3' for CCL13; 5'-GGA CTT CCT GGA TCC TCC TC-3' and 5'-AGC AGT CAG CAG CAA AGT GA-3' for CCL15; 5'-ATG GCC CTG CTA CTG GCC CTC AGC CTG-3' and 5'- TTA ACT GCT GCG GCG CTT CAT CTT GGC-3' for CCL19; 5'-TGT AGG GCG ACG GTT TTA-3' and 5'-TCC ACC ACA ACA TGC AG-3' for CCL25; 5'-GGC CCT GCC CTT ATA GC-3' and 5'-CTA ACT TGG GGT TGA CAT T-3' for CXCL1-3; 5'-TGT TGA GAG AGC TGC G-3' and GGG TTC AGA GAC CTC CA-3' for CXCL5; 5'-GAA GTG GTA GCC TCC C-3' and 5'-GCT TTC CCC CAC ACT C-3' for CXCL6; 5'-TCC GCT GCA TGT GTA TAA AG-3' and 5'-ATA GGT ATC CTG AAT AAA TGA GAA C-3' for CXCL7; 5'-CAT GCT GGT GAG CCA AGC AGT TTG AA-3' and 5'-CAC TTC TGT GGG GTG TTG GGG ACA AG-3' for CXCL9; 5'- CGA TGC CTA AAT CCC AAA TCG AAG CA-3' and 5'-AAT TGC TGG ACT CCT TTG GGC AGT GG-3' for CXCL11; 5'-ATG AAC GCC AAG GTC GTG GTC-3' and 5'-TGG CTG TTG TGC TTA CTT GTT T-3' for CXCL12; 5'-TCT CTC CAG GCC ACG GTA TTC-3' and 5'-ACC ATT TGG CAC GAG GAT TCA C-3' for CXCL13. CCL21 primer was purchased from Biosource (Camarillo, CA) and CCL2, CCL5, CXCL8 and CXCL10 primers were purchased from R&D Systems. PCR reactions were performed for 35 cycles (94°C, 45 sec; 60°C for CCL4, CCL19, CCL21, CXCL1, CXCL5, CXCL6, CXCL7, CXCL9, and CXCL11; 56°C for CXCL12, 55°C for CCL2, CCL3, CCL5, CCL25, CXCL8 and CXCL10; 52°C FOR CCL7 and CCL15; 48°C for CCL8, CCL11 and CCL13, 45 sec; 72°C, 45 sec) using the SuperScript One-Step RT-PCR kit (Invitrogen). All PCR involved an initial denaturation step at 94°C for 45 sec to 1.5 min and a final extension step for 7 min at 72°C. All PCR products were analyzed using 10% novex-TBE gel (Invitrogen). Supernatants obtained from CRC cells on day 6 of culture were tested for the presence of CCL2, CCL3, CCL15, CCL19, CCL21 and CXCL11 using ELISA kits (R&D Systems).

### Phenotyping

Phenotyping of tumor cells and T cells was performed as described [32]. In brief, cultured cells were incubated with saturating concentrations of FITC or PE-conjugated mAb (5 μg/ml) detecting human lymphocyte and tumor markers in FACS buffer for 1 h at 4°C, followed by excess mAb removal by washing in FACS buffer. Binding of the mAb was analyzed as described [32].

### T-cell migration in organotypic CRC culture (reconstruct)

Organotypic CRC cultures were initiated as described [32]. In brief, 1.8 × 10^5 ^fetal fibroblast cells were mixed with collagen matrix (450 μl) and plated in a 24-well tissue culture treated plate (Corning, Corning, NY). After 24 h, WC007 CRC cells (1 × 10^5^) were seeded on top of the collagen layer and after 24 h, a separating layer of fibroblasts in collagen matrix (100 μl, 500 μm) was added on top of the CRC cells. CTLs (1-10 × 10^5^) were mixed with fibroblasts (1 × 10^5^) in 250 μl collagen matrix and plated on top of the separating layer. In some cultures, CRC cells were stained with CellTracker Blue CMAC (15 μM, for 40 min at 37°C; Invitrogen) and CTL were pre-stained with CFDA-Green (5 μM, Invitrogen). For control reconstruct, autologous PHA blasts (PBMC stimulated with PHA [1% v/v, Invitrogen] and propagated in recombinant interleukin (IL)-2 [20 U/ml; a gift from the Biological Resources Branch, National Cancer Institute-Frederick Cancer Research and Development Center, Frederick, MD] for 3-4 weeks) were used. Reconstructs were incubated in medium (50% DMEM, 50% CRC medium supplemented with 2% human AB serum). On various days after addition of T cells reconstructs were fixed and processed for histological evaluation as described earlier [32]. The percentage of apoptotic tumor cells was determined by counting apoptotic nuclei and intact tumor cells in sections stained with H&E.

### Blocking of T-cell migration in reconstruct

T-cell migration in the reconstruct was performed in the presence of anti-chemokine or chemokine receptor Abs (10 μg/ml) or isotype-matched control Ab added above the separating layer, followed by addition of CTL-fibroblast collagen layer [32]. To evaluate whether excess chemokine can block migration of T cells, the chemokine (50 ng/ml) was added into the medium on top of the T-cell layer. The percentage of apoptotic tumor cells in the presence and absence of inhibitor was determined and the percentage of inhibition of apoptosis by Abs or chemokines was calculated [32].

### Chemotaxis assay

CTL migration was evaluated using a 24-well, Transwell plate (8.0-μm pore size; Corning, Corning, NY) as described earlier [34]. In brief, T cells were washed once with RPMI1640 medium, cell count re-adjusted (5 × 10^5 ^cells/mL) in T cell medium [33] and an aliquot (100 μL) of T-cell suspension was placed in the top chamber of the Transwell. Bottom chamber of Transwell plate received chemokine (500 μL in T cell medium) at the indicated concentration prior to the addition of T cells in the top chamber. After 90 min incubation at 37^o ^C in a 5% CO2 atmosphere, the top chamber was removed, and the number of T cells that had migrated into the bottom chamber was counted under the microscope.

### Immunohistochemistry

Formalin-fixed paraffin-embedded sections (5 μm) were deparaffinized by sequential application of Sub-xylene substitute (3 × 10 min, Surgipath Medical Industries, Richmond, IL) and re-hydrated through a graded series of ethanol after which they were rinsed shortly in phosphate buffered saline (PBS). In situ end labeling (ISEL) was performed as described earlier [35]. Briefly: sections were air-dried and digested with proteinase K (1.25 μg/ml in 50 mM Tris-HCl, 1 mM EDTA, pH 8.0) for 30 min at 40°C in a humidified chamber. After incubation, sections were rinsed in distilled water followed by sequential changes of ethanol (70-95%), and air dried. Sections were end-labeled by incubating with biotinylated dCTP, dATP, and non biotinylated dTTP, dGTP (0.01 mM each, Invitrogen) in the presence of DNA polymerase Klenow I fragment (Promega Corporation, Madison, WI) for 60 min at 40° C. Sections were blocked later for endogenous peroxidase activity with H_2_O_2 _(0.3% in methanol, for 20 min at RT, serially rinsed with distilled water and PBS) and the biotin-labeled DNA sequences were detected by horseradish peroxidase (HRP)-conjugated streptavidin for 40 min at 37° C. Slides were washed and incubated with 2', 5'-diaminobenzidine (DAB, Vector Laboratories, Burlingame, CA) followed by counterstaining with hematoxylin. Caspase staining was performed using an Ab specific for the active form of caspase 3 (present only in cells undergoing apoptosis). In brief, sections were blocked for endogeneous peroxidase as above, followed by addition of avidin/biotin and protein blocking using respective blocking kits (Vector Laboratories and Immunotech). After blocking, slides were incubated with rabbit anti-human active caspase 3 polyclonal Ab (1:1500 dilution, R & D Systems), at 4°C overnight, followed by incubation with biotinylated anti-rabbit Ab and HRP-conjugated streptavidin (both from Vector Laboratories). Signals were visualized with 2', 5' DAB as the substrate. The slides were counterstained with hematoxylin. Normal rabbit gamma globulin was used as a negative control (MP Biomedical Services, Santa Ana, CA).

### Statistical analyses

Differences between experimental and control values were analyzed for significance by 2-sample Student's *t*-test.

## Results

### Functional characteristics of CTL007 in reconstruct

**Figure 1:**
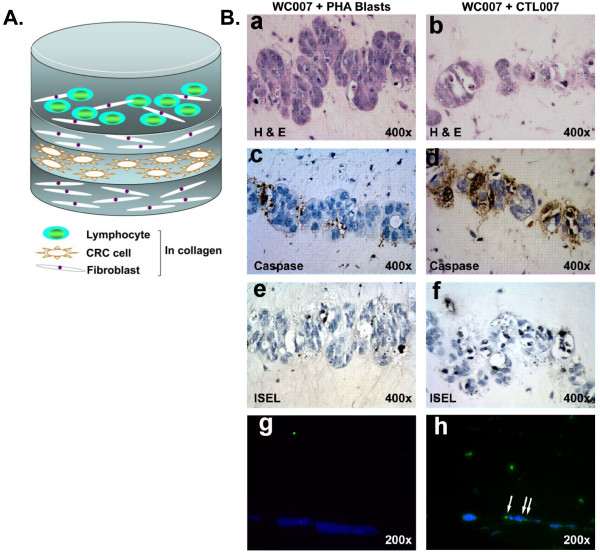
**Migration of CTL007 toward WC007 CRC cells in the reconstruct**. A. Reconstruct schema. B. [a-f]: The bottom layer of reconstructs contained 1.8 × 10^5 ^fibroblasts in 450 μl type I collagen gel which was followed by addition of CRC cells (1 × 10^5^) on top after 24 hr. After further 24 hr, a separating layer of fibroblasts in collagen gel (100 μl, 500 μm) was added on top of cancer cells, followed by the top layer containing CTL (1 × 10^5 ^[32]), or autologous PHA blasts (control lymphocytes [a, c, e]) mixed with 1 × 10^5 ^fibroblasts and collagen. Reconstructs were harvested on day 6 (3 days after adding T cells), fixed in buffered formalin and embedded in paraffin. a, b: Staining with H&E. c, d: Specific brown staining of apoptotic cells by anti-caspase 3 Ab. e, f: In situ end-labeling (ISEL); black staining of nuclei of cells undergoing apoptosis. Apoptosis was significantly higher in presence of CTL (d and f) than in presence of autologous PHA blasts (c and e; p < 0.0005). g-h: Reconstructs were prepared as in a-f, but tumor cells were stained with CellTracker Blue CMAC; autologous PHA blasts (g) and CTL007 (h) were pre-stained with CFDA-Green. Reconstructs were harvested on day 4 (2 days after adding T cells), and sections were photographed in the Nikon fluorescence microscope using appropriate filters. Arrows indicate binding of CTL007 to the tumor cells.

**Figure 2:**
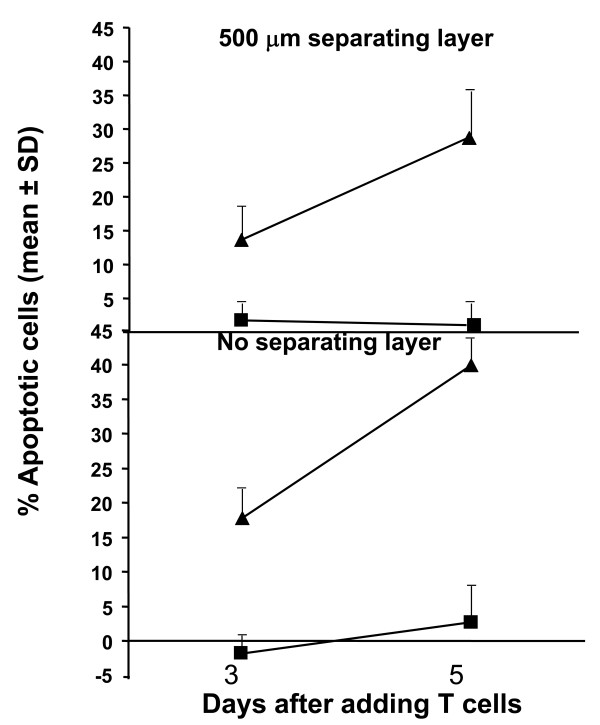
**Time course of WC007 CRC apoptosis induction by CTL007 in reconstruct**. Reconstructs were prepared as in Fig.1. An E: T ratio of 2:1 was used in this assay, reconstructs harvested on day 6 or 8 (3 or 5 days after adding T cells), fixed, processed and enumerated as described in Fig.1. Values represent mean percentage of apoptosis/field (total of 10 fields), of cultures with lymphocytes corrected by the value obtained without lymphocytes, ± SD (bars). Percent apoptotic tumor cells of reconstruct cultures with CTL (▲) were significantly higher than cultures with PHA blast (■) on both days, for cultures with and without separating layer (at p < 0.05 level). Percent apoptotic tumor cells of reconstruct cultures was significantly (p = 0.005) higher on day 5 than on day 3 for cultures with and without separating layer. The separating layer had no significant effect on percent apoptotic cells.

We have shown that CTL007 specifically lyses autologous WC007 colon carcinoma target cells in an HLA-class I (A1) restricted manner and does not have any inhibitory T cell function [33]. Studies of CTL007 in the reconstruct showed that these CTL induce tumor cell apoptosis. Apoptosis of WC007 cells was determined microscopically in H&E-stained cultures, and by histochemistry (Figure 1B [a-f]). Reconstruct containing autologous PHA blasts has large numbers of healthy WC007 tumor cells (Figure 1B [a]). In contrast, culture established with CTL007 shows greater proportion of dead tumor cells (Figure 1B [b]) which was further confirmed by apoptosis assays [caspase staining] (Figure 1B [c and d]) and in situ end labeling (ISEL; Figure 1B [e and f]). Tumor cell apoptosis was quantified microscopically by enumerating apoptotic tumor cells in H&E-stained cultures (Table 2). The CTL induced significant apoptosis in the autologous CRC cells, as compared to reconstructs with tumor cells alone or tumor cells plus PHA blasts. The percentage of apoptotic WC007 cells depended on E: T ratios in the reconstruct (Table 2). CTL007 showed significantly (p = 0.005) higher lytic capacity on day 5 of lymphocyte culture compared to day 3, irrespective of the presence or absence of a separating collagen/fibroblast layer (Figure 2). Thus, longer exposure of tumor cells to CTL007 results in increased apoptosis of tumor cells.

**Table 2:** Apoptosis induction by CTL007 in reconstruct with autologous WC007 CRC cells*

Lymphocytes	E:T	Total number of tumor cells mean ± SD/field (10 fields)	Number of apoptotic tumor cells mean ± SD/field (10 fields)	Percentage of apoptotic cells, mean ± SD/field (10 fields)
Experiment I				
CTL007	1:1	80.5 ± 38.8	23.5 ± 8.7	31.7 ± 11^c, d^
PHA blast	1:1	136.2 ± 54.8	15.9 ± 6.8	12.1 ± 3.9^c^
No lymphocytes	NA	90.1 ± 3.5	7.1 ± 2.8	8.1 ± 3.5^d^

Experiment II				
CTL007	10:1	116.7 ± 19.4	94.8 ± 19	79.5 ± 6.7^a, b^
PHA blast	10:1	118.3 ± 23.6	34.7 ± 11.8	27.5 ± 4.7^a^
No lymphocytes	NA	121.1 ± 22.3	25.8 ± 10.7	21.3 ± 10.1^b^

^a-d ^Values with the same symbol differ significantly from each other (Student's 2-sided t-test; p < 0.0001.

* Reconstructs were established with a separating collagen-fibroblast layer, CTL007 or autologous PHA blasts were added onto the top layer. Reconstructs were harvested either on day 3 (experiment I) or day 4 (experiment II) after adding T cells. Day of harvesting of reconstruct cultures was based on optimum constriction of the collagen gel of reconstruct cultures. The ratio of apoptotic tumor cells was determined by counting apoptotic nuclei and intact tumor cells in sections stained with H&E. Data represented are from two independent experiments.

Migration of CTL was visualized in reconstructs using T cells labeled with CFDA-Green and tumor cells labeled with CMAC-Blue. Lymphocytes migrated from the top layer of collagen and fibroblasts through a separating layer of collagen and fibroblasts toward WC007 tumor cells (Figure 1B [h]). Although a small proportion of PHA blasts also migrated to the tumor cell layer, the PHA blasts did not induce significant apoptosis in the tumor cells (Figure 1B [a, c, e, g; Table 2).

### Phenotypic characteristics of CTL007 and WC007 CRC cells

CTL007 and WC007 CRC cells were phenotyped with special emphasis on molecules that might be involved in the interactions of these cells with each other and components of the reconstruct (Table 3). CTL007 is a CD4+ (> 96%) T cell line that expresses several adhesion and co-stimulatory molecules as described in Table 3. It also expresses α2 (CD49b) and β1(CD29) integrins (Table 3) that are important for T-cell interaction with collagen in the reconstruct, which might result in T-cell activation [36,37]. In addition, T cells also express LFA-1a (CD11a), ICAM-1 (CD54), and CD44 (Table 3) and these molecules facilitate interaction of the lymphocytes with fibroblasts in the reconstruct [38,39]. This interaction results in the activation of both lymphocytes and fibroblasts through secretion of growth and survival factors, cytokines, and fibronectin [38-41].

**Table 3:** Phenotypic and functional markers of anti-CRC CTL007 and autologous WC007 tumor cell line

Parameter investigated^a^	Cell lines (% cells positive)	
	
	CRC WC007	CTL007
HLA Class I	99.8	95.6
HLA Class II	< 1	60
CD4	NA	96
CD8	NA	< 1
CD25	< 1	30
CD40L	2.1	13
CD44	48	79.7
CD80 (B7-1)	85	< 1
CD49a (α1 integrin)	48.2	26.1
CD49b (α2 integrin)	63.2	32.5
CD29 (β1 integrin)	56.9	79.8
CD95 (FAS)	65.1	95.1
CD95L (FASL)	19	< 1
CD54 (ICAM-1)	84.5	89.3
CD11a (LFA-1a)	< 1	84.2

NA = not applicable

^a^All markers were determined by flow cytometry and data represented are from a single experiment and the Results were confirmed in at least two independent experiments.

WC007 CRC cells express both HLA class I and II molecules, FAS, ICAM-1, and various integrins. Expression of α2 and β1 integrins by the CRC cells may facilitate their binding to collagen [42]. CRC cells also express FAS ligand (< 19%) and are positive for B7-1 and ICAM-1. B7-1 and ICAM-1 on the CRC cells potentially interact with CD28 and LFA-1a on the CTL, respectively, which may result in T-cell stimulation [43].

Thus, several phenotypic markers are expressed by CTL007 and WC007, which are known to facilitate interactions between these cells and between the lymphocytes or tumor cells and collagen or fibroblasts in the reconstruct, leading to activation of T cells as well as T-cell migration toward tumor cells.

### Chemokine and chemokine receptor involved in CTL007 migration toward WC007 cells

CTL007 express the chemokine receptors CCR1, CCR2, CCR3, CCR5, CCR7, CCR9, CXCR1, CXCR2, CXCR3, CXCR4, and CXCR5 (Table 4). For each chemokine receptor, with the exception of CCR3, CXCR1, CXCR2, CXCR4, and CXCR5 the corresponding chemokine(s) was expressed by WC007 CRC cells, as determined by RT-PCR and protein expression confirmed by ELISA (Table 4).

**Table 4:** Chemokine receptors expressed by CTL007, and chemokines produced by WC007 CRC cells

Chemokine receptors expressed by CTL007^a^		Chemokines		
**Chemokine receptors**	**% positive** **cells**	**Known to bind** **to receptor**	**Expressed by WC007^b^**	
			
			**RT-PCR**	**ELISA (pg/ml)**

CCR1	5.7	CCL3	+	< 30^c^
		CCL5	-	ND^d^
		CCL7	-	ND
		CCL13	-	ND
		CCL15	+	35
CCR2	65	CCL2	+	51.6
		CCL7	-	ND
		CCL8	-	ND
		CCL13	-	ND
CCR3	19.4	CCL7	-	ND
		CCL8	-	ND
		CCL11	-	ND
		CCL13	-	ND
		CCL24	-	ND
CCR5	10.1	CCL3	+	< 30^c^
		CCL4	+	16
		CCL5	-	ND
CCR7	9.5	CCL19	+	< 20^c^
		CCL21	+	< 30^c^
CCR9	16.4	CCL25	-	ND
CXCR1	16.7	CXCL6	-	ND
		CXCL8	-	ND
CXCR2	15.4	CXCL1	-	ND
		CXCL5	-	ND
		CXCL6	-	ND
		CXCL7	-	ND
		CXCL8	-	ND
CXCR3	16.9	CXCL9	-	ND
		CXCL10	-	ND
		CXCL11	+	35.6
		CXCL13	-	ND
CXCR4	17.6	CXCL12	-	ND
CXCR5	32.4	CXCL13	-	ND

^a ^Chemokine receptor expression as determined by FACS and the following chemokine receptors were not expressed by CTL007:CCR4, CCR6, CCR8, CCR10, CX3CR1

^b ^Chemokine expression was detected by RT-PCR and if they were positive in RT-PCR, then protein expression was confirmed by ELISA. CCL3, CCL19 and CCL21 mRNA were detected in WC007 cells by RT-PCR, but the protein expression was below detection limit of

ELISA. Data represented are from a single experiment and the Results were confirmed in at least two independent experiments.

^c^below detection limit

^d^ND-Not determined

**Figure 3:**
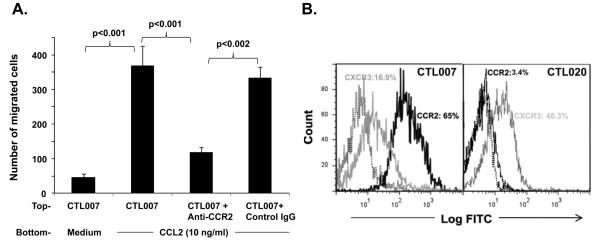
**A. CTL007 migrates toward CCL2 in Transwell**. T cells were placed in the top chamber of the Transwell. CCL2 (10 ng/ml) in T cell medium, or T cell medium alone were added to the bottom of the Transwell (duplicate wells). After 90 min of culture, the number of migrated cells in the bottom chamber was counted under the microscope. Some T cell cultures were pre-incubated and treated with saturating concentration (10 μg/ml) of mouse anti-CCR2 Ab or mouse IgG as control. B. Expression of CCR2. T cells grown in log phase were incubated with either anti-CCR2 or anti-CXCR3 Ab (black or grey solid line) or mouse IgG control (dotted line) in RPMI 1640 with 5% human AB serum for 1 h at 4°C. After washing, FITC-labeled anti-mouse IgG was added. Expression of chemokine receptors was detected by flow cytometry.

We evaluated possible roles of chemokine receptors CCR1, CCR2, CCR3, CCR5, CCR7 and CXCR3 expressed by the T cells in the migration of the CTL cells toward CRC cells in the reconstruct. T-cell migration was measured as a function of tumor cell apoptosis and not absolute number of T cells at the tumor cell layer, since T cells may themselves undergo apoptosis after inducing tumor cell apoptosis and one T cell may induce apoptosis in more than one tumor cell. Only apoptotic tumor cells, and not T cells, were counted. Apoptotic tumor cells could be distinguished from apoptotic T cells based on size difference. However, evaluation of apoptotic tumor cells does not allow us to distinguish between T cells with high migratory and low lytic activity and T cells with low migratory and high lytic activity. Nevertheless the ratio of apoptotic tumor cells correlates with CTL migration. Blocking of chemokine receptor CCR2 but not CCR1, CCR3, CCR5, CCR7 or CXCR3 on CTL007 with Abs significantly inhibited tumor cell apoptosis (Table 5). To determine the involvement of CCR2 ligand (CCL2) in T cell migration and apoptosis, excess recombinant CCL2 or anti-CCL2 Ab was added to the top of the T-cell layer or to the separating layer. Both excess CCL2 and anti-CCL2 Ab were able to inhibit T cell migration and tumor cell apoptosis significantly (p < 0.05, Table 5). Involvement of CCR2 and CCL2 in induction of migration of CTL007 was further confirmed in chemotaxis assay using Transwell plates (Figure 3). Treatment of T cells with anti-CCR2 Ab significantly inhibited (p < 0.001) the migration of CTL007 toward recombinant CCL2, whereas control IgG treatment of T cells had no effect (Figure 3). These data further confirm of our finding that CCR2 receptor on T cells and CCL2 secreted by tumor cells are involved in migration of T cells towards tumor cells. Predominant expression of CCR2 (65%) when compared to other chemokine receptors (most <20% except CXC5; Table 4 Figure 3B) further supports the role of CCR2 in T cell migration. Relative high expressions of CCR2 (65%) on CTL007 and CXCR3 (48.3%) on CT020 (Figure 3B) respectively suggests dominant expression of certain chemokine receptors (CCR2 for CTL007 and CXCR3 for CTL020) may be a decisive factor in migration of lymphocytes.

**Table 5:** Induction of tumor apoptosis by CTL007 is inhibited by anti-CCR2 and CCL2 Abs and excess CCL2a

Blocking agent	Number of tumor cells/field (10 fields)	Number of apoptotic cells/field (10 fields)	% of apoptotic cells	% of tumor cell apoptosis inhibition
None	21.8 ± 9.7	8.2 ± 9	40.5 ± 9.7^b^	-
Control IgG	21 ± 2.2	8.4 ± 1.1	40.1 ± 4.9^c^	-
Anti-CCR1 Ab	22 ± 3.4	10.0 ± 1.6	45.6 ± 5.0	-13.7
Anti-CCR2 Ab	19.6 ± 1.1	2.6 ± 0.5	13.2 ± 2.3^c^	67.1
Anti-CCR3 Ab	22.4 ± 2.8	9.2 ± 1.9	40.7 ± 4.2	1.5
Anti-CCR5 Ab	21 ± 2.6	9.6 ± 1.8	45.5 ± 3.8	-13.4
Anti-CCR7 Ab	18.8 ± 2.4	7.2 ± 1.6	38.0 ± 4.1	5.2
Anti-CXCR3 Ab	21.4 ± 1.5	9.2 ± 0.8	43.1 ± 1.8	-7.5
CCL2	21.4 ± 4.4	3.6 ± 1.1	17.2 ± 6.1^b^	57.5
Anti-CCL2 Ab	17.8 ± 2.6	2.2 ± 0.4	12.7 ± 3.8^c^	68.6

^a ^Reconstructs consisted of a bottom layer of collagen and fibroblasts, followed by a tumor cell layer and a separating layer of collagen and fibroblasts. Anti-chemokine receptor or control Abs were added, followed by a top layer containing CTL mixed with fibroblasts and collagen (E: T = 3:1). Excess CCL2 (50 ng/ml) was added to the T-cell layer. Percentage of apoptotic tumor cells in 6 day cultures was determined. ^(b, c)^Values with same letter differ significantly from each other (p < 0.05). Data represented are from a single experiment and the Results were confirmed in at least two independent experiments.

### CCR2 and CCL2 expression by T cells and tumor cell lines, respectively, derived from additional CRC patients

We investigated the distribution of CCR2 and CCL2 in T cells and tumor cell lines, respectively, established from specimens of additional CRC patients. Expression of CCR2 receptor and its ligand by the cells of additional CRC patients would suggest that involvement of the receptor/ligand in T-cell migration toward tumor cells may not be a unique observation made in a single CRC patient, but may be found in other patients. Ten tumor reactive T-cell lines derived from TIL of additional CRC patients were analyzed for CCR2 expression and four of these showed CCR2 expression (Table 1). Furthermore, two of the established CRC cell lines produced CCL2 (data not shown).

## Discussion

We have demonstrated here that CTL007 migrate through a 500 μm collagen/fibroblast separating layer toward tumor cells, resulting in tumor cell apoptosis. We have also shown that migration is dependent on CCR2 expressed by T cells and CCL2 secreted by tumor cells.

Our recently developed novel three-dimensional culture system offers a unique way of studying migration of leukocytes toward tumor cells and the factors that influence leukocyte migration under physiological conditions [32,34]. As described in our previous studies, human CRC is grown *in vitro *under three-dimensional conditions using a mixture of collagen and fibroblasts [32,44]. Interaction of α2 and β1 integrins on CRC-specific T cells with collagen and the presence of activated fibroblasts help to maintain Ag-specific T cells in a state of activation in absence of exogenous addition of IL-2 [36,37]. In addition, T cells could interact with fibroblasts via adhesion molecules like LFA-1a, ICAM-1 and CD44 which could in turn stimulate fibroblasts to secrete inflammatory cytokines such as IL-1, IL-6, IL-7 [38,39], and fibronectin [41]. IL-1 could stimulate T cells to express IL-2 receptor and induce secretion of IL-2 [45]. IL-6 and IL-7 are T-cell survival factors [46], and fibronectin stimulates predominantly resting lymphocytes [41]. Other investigators have used collagen matrices to study interaction of leukocytes with tumor cells, but they have not demonstrated CTL migration resulting in tumor cell apoptosis in a culture system similar to the reconstruct cultures shown here [47-49].

In the present study, CCL2 produced by CRC cells attracts CTL through binding of CCL2 to its corresponding chemokine receptor CCR2 on the T cells. This was demonstrated by blocking of T-cell migration in presence of addition of excess chemokine CCL2 in the T-cell layer of the reconstruct or the addition of Abs to CCL2 or CCR2, each applied on top of the separating collagen/fibroblast layer. Involvement of CCR2 in T-cell migration was further confirmed in chemotaxis assay in Transwell plate experiment by treating T cells with anti-CCR2 Ab. Although CTL007 has several chemokine receptors matching chemokine produced by CRC cells, predominant expression of CCR2 (65%) by the T cells and the relatively higher amount of CCL2 secretion by tumor cells may have been a decisive factor in T-cell migration.

Many carcinomas, including breast, colorectal, pancreatic and renal carcinomas, and neuro-ectodermal tumors such as melanomas, medulloblastomas, neuroblastomas and glioblastomas are known to produce CCL2 (reviewed in [1]; [20-22,50]. CCL2 secretion by tumor cells can aid in tumor progression, angiogenesis and metastasis [1,5,10]. Also, the secretion of CCL2 by tumor cells results in infiltration of tumor cells by leukocytes including T cells, NKT cells and macrophages (reviewed in [1]; [20-22]). To our knowledge, the role of CCL2-dependent T-cell migration in CRC is largely unknown. In mouse tumor model systems, melanoma cells secreting high amounts of CCL2 attract macrophages resulting in inhibition of tumor growth. However, tumor cells secreting low amounts of CCL2 promote tumor growth by stimulating angiogenesis [23]. In another study, tumor cells transfected with CCL2 showed decreased metastasis due to increased infiltration of macrophages and susceptibility of tumor cells to lysis by infiltrating macrophages [24]. Thus, patients may be vaccinated with chemokine-transduced tumor cells [51] or tumor-associated Ags fused to chemokines [52]; alternatively, patients may be treated with anti-tumor Ab/chemokine fusion protein, which may attract adoptively transferred lymphocytes to the tumor area [12,52,53]. In light of the mouse study by Nesbit et.al., [23], one needs to carefully modulate the expression of CCL2 to attract immune cells toward tumor cells. Thus, chemokines may be useful for immunotherapy of cancer patients.

In addition to therapeutic implications, the results of our study have prognostic potential. Infiltration of CRC with T lymphocytes is correlated with a favorable prognosis [54], and chemokine receptor expression by T lymphocytes as well as chemokine production by tumor cells should be explored for their possible association with prognosis. Identification of tumor cells secreting CCL2 or T-cells expressing CCR2 in the tumor microenvironment could be a useful prognostic marker in CRC patients [17,18,22,55-58]. In our earlier study, we have shown that migration of CTL derived from a CRC patient towards autologous tumor cells was mediated by CXCR3 expressed by the T cells, and CXCL11 chemokine secreted by the autologous tumor cells [32]. In the present study, we show that migration of CTL is dependent on CCL2 and CCR2. Presence of CCR2 in T cells obtained from four of the ten additional patients suggests that CCR2 expression may not be that uncommon. It is likely that in each individual CRC patient a unique chemokine/chemokine receptor pair might be involved in attraction of T cells towards tumor cells. Hence, it is essential to identify the chemokine/chemokine receptor pair responsible for attraction of T cells towards tumor in each CRC patient for individualized therapy.

## Conclusions

Our study demonstrates the role of CCR2 and CCL2 in migration of CTLs towards tumor. Data obtained from additional CRC patients further strengthen the role of CCR2 and CCL2 in lymphocyte migration. Identification of chemokine/chemokine receptor pair responsible for attraction of T cells towards tumor in each patient will aid individualized therapy approaches using gene modification of tumor cells with chemokine or chemokine/anti-tumor Ab fusion proteins to attract CTLs that potentially could inhibit tumor growth.

## Competing interests

The authors declare that they have no competing interests.

## Authors' contributions

KB carried out reconstruct studies and all the data analyses; PR carried out histological staining, RT-PcR of chemokines; TZ performed ELISA assays for chemokines; LG cultured and expanded CTLs for the assay, and characterized chemokine receptor expression on T cells; MM and NM recruited patients for the study, DH and RS designed the study and in coordination with all others drafted the manuscript. All authors read and approved the final version of the manuscript.
